# Hypothermia in the intensive care unit after surgery: a registry based retrospective cohort study

**DOI:** 10.1016/j.bjao.2026.100556

**Published:** 2026-08-13

**Authors:** Jeremy Weiss, Matthew Durie, Tamishta Hensman, Tess Evans, David Pilcher

**Affiliations:** 1 Department of Intensive Care, Austin Hospital, Heidleberg, Australia; 2 Intensive Care Unit, Royal Melbourne Hospital, Parkville, Australia; 3 Department of Anaesthesia and Pain Medicine, Royal Melbourne Hospital, Parkville, Australia; 4 Department of Critical Care, Melbourne Medical School, University of Melbourne, Parkville, Australia; 5 Intensive Care Unit, Victorian Heart Hospital, Clayton, Australia; 6 Australian and New Zealand Intensive Care Society (ANZICS) Centre for Outcome and Resource Evaluation, Prahran, Australia; 7 The Alfred Intensive Care Academic Centre, Bayside Health, Prahran, VIC, Australia; 8 Australian and New Zealand Intensive Care Research Centre, School of Public Health & Preventive Medicine, Monash University, Prahran, Australia

**Keywords:** hypothermia, intensive care, perioperative care, postoperative hypothermia, registry, temperature management, epidemiology

## Abstract

**Background:**

Hypothermia is common in patients admitted to the ICU after surgery; however, its relationship with postoperative outcomes has not been fully explored. This study investigates the association between postoperative hypothermia in the ICU and patient mortality.

**Methods:**

Retrospective cohort study using the Australian and New Zealand Intensive Care Society adult patient database. Postoperative noncardiac surgical ICU admissions between 2018 and 2023 were included. Hypothermia was defined as temperature <36°C. The lowest temperature recorded within the first 24 hours of ICU admission was used. The primary outcome was in hospital mortality with secondary outcomes including ICU mortality rate, length of stay and ICU readmission rates. Descriptive and multivariable logistic regression models were used to investigate the relationship between postoperative temperature and mortality.

**Results:**

Of 425,648 postoperative admissions to the ICU, 165,111 (38.8%) experienced hypothermia within 24 h of their admission. Patients with hypothermia had higher hospital mortality rates of 4.3%, compared with 2.1% in the normothermia group (adjusted odds ratio 1.34; 95% confidence interval 1.28–1.39). Mortality rates increased with lower temperatures with the 32–32.9°C group experiencing the highest mortality rate (adjusted odds ratio 3.03; 95% confidence interval 2.30–4.00). ICU readmission rates and hospital and ICU length of stay were similar between groups.

**Conclusions:**

In this retrospective cohort study we report that ostoperative hypothermia in the ICU setting is common in Austrailia and New Zealand and was associated with an increased mortality rate. Early recognition and prevention of hypothermia in the intensive care setting may be a modifiable risk factor to reduce mortality for this patient cohort.

**Keypoints**

• The incidence of postoperative hypothermia in the adult ICU setting is common.
• This retrospective cohort study explores the relationship between hypothermia after noncardiac surgery and ICU outcomes in Australia and New Zealand hospitals.
• Between 2018 and 2023, 39.4% of patients had a recorded temperature <36 degrees Celsius; meeting the definition for hypothermia.
• Mortailty rates were double in the hypothermia group compated to the non-hypothermic group (adjusted odds ratio of 1.34) with mortality rates higher for the lowest temperatures.
• Postoperative hypothermia in those adult pateints requireing ICU admission may be ether a modifable risk factor or a marker of risk.

Hypothermia is common in patients admitted to the ICU after surgery.^1^ Previous studies have shown an incidence of early (within 24 h) postoperative hypothermia in ICU between 35% and 60%.^1^^,^^2^ The causes of hypothermia in these patients may include convective exposure intra-operatively, anaesthetic agents that inhibit the thermoregulatory system, intra-operative blood loss and/or the use of cold intravenous fluids.^3^, ^4^, ^5^ Whilst some operative specialties such as cardiac surgery may rely on inducing hypothermia for circulatory arrest, in noncardiac surgery cases it is usually viewed as an unintended consequence.^6^

The potential adverse physiological effects of hypothermia in postoperative patients are numerous and include impaired wound healing, increased incidence of postoperative infection, bleeding and cardiac arrhythmia.^7^, ^8^, ^9^, ^10^ Risk factors for inadvertent perioperative hypothermia include prolonged surgery, extremes of age, low Body Mass Index (BMI) and emergency major surgery.^11^^,^^12^ Hypothermia prolongs postoperative anaesthetic recovery time and places a significant added cost on the healthcare system.^13^^,^^14^ Whether these adverse risks affect patient-centred outcomes in the ICU after surgery remains an unresolved topic of interest. A 2013 retrospective analysis of postoperative hypothermia in the ICU after elective noncardiac surgery found hypothermia to be highly prevalent (46%).^1^ The median lowest temperature was 35.9°C across all groups and multivariable analysis did not demonstrate a mortality difference in patients with transient or persistent hypothermia; odds ratio (OR) 1.07 (0.96–1.20) and 1.50 (0.96–2.33) respectively.

This study presents a contemporary analysis of hypothermia in a large cohort of postoperative ICU patients with and without adjustment for known covariables. We tested the hypothesis that hypothermia may be associated with an increased risk of mortality in patients admitted to ICU after noncardiac surgery and that this effect may be proportional to the degree of hypothermia.

## Methods

### Design and setting

We performed a retrospective observational cohort study using data prospectively collected routinely from all ICUs contributing data to the Australia and New Zealand Intensive Care Society Adult Patient Database (ANZICS-APD) between January 2018 and June 2023.

### Participants

Patients included in this study were age 18 or older, admitted after surgery to participating ICUs during the study time period either on elective or emergent basis. Exclusion criteria included patients who had undergone cardiac surgery and patients admitted for palliation or potential organ donation. As this study was comparing normothermia and hypothermia groups, patients with persistent hyperthermia (a lowest temperature that was 38°C or above in the first 24 h) were excluded as they would represent an additional clinical category outside the scope of this study. Only the first ICU admission episode was included where patients were readmitted to the intensive care within the same hospitalisation episode.

### Data source

The ANZICS Adult Patient Database (APD) is a binational registry dataset collected for benchmarking and quality assurance purposes by the ANZICS Centre for Outcomes and Resource Evaluation (CORE). It includes deidentified individual patient data from 98% of ICUs in Australia and 68% of ICUs in New Zealand. Data includes patient demographics, admission diagnoses, acute physiology within 24 h of ICU admission, chronic health conditions, source of ICU admission, status of elective or emergency surgery and outcomes such as length of stay and mortality.^15^ Temperature data used in our study are the recorded lowest and highest core temperatures. The registry does not specify which site of core temperature measurement has been used and may include peripheral temperature if core temperature was unable to be obtained.

This project was approved as a low-risk study, with waiver of individual patient informed consent, by the Human Research Ethics Committee of the Alfred Hospital Project 421/24. The ANZICS CORE Management Committee approved access to the data in accordance with standing protocols.

Patients were categorised as those with hypothermia and normothermia based on the lowest recorded temperature within 24 h of admission to ICU. For this study, hypothermia was defined as lowest recorded temperature of <36°C and normothermia was 36–37.9°C. An additional comparison was made stratifying patients into temperature categories of 1°C intervals with patients <32°C aggregated as a single group.

### Outcomes

The primary outcome was in-hospital mortality. Secondary outcomes included ICU mortality, hospital length of stay, ICU length of stay and ICU readmission rates. To account for differing pre-ICU periods, hospital length of stay was considered to begin at ICU admission when the temperature data were recorded.

### Statistical analysis

Categorical data were summarised as count (percentage) and compared using the χ^2^ test. Mortality was also reported using Kaplan–Meier plots displaying time to death in hospital or discharge. Continuous data were tested for normality and found to be skewed. These are summarised as median (interquartile range) and compared using the rank sum test for non-parametric data. A *P*-value of <0.05 and 95% confidence intervals (CIs) were used to evaluate statistical significance. Multivariable logistic regression analyses were performed with temperature categorised in two groups (hypothermia *vs* normothermia), in 1°C temperature strata and also using restricted cubic splines. Analyses were adjusted for age, sex, illness severity, elective *vs* non-elective surgery, field of surgery, frailty, invasive ventilation in first 24 h of ICU admission, treatment limitation and pre-ICU hours. Frailty was defined using modified Rockwood Dalhousie Clinical Frailty Scale (CFS) with removal of the 9^th^ level of scoring (Terminally Ill).^16^ CFS of ≥5 was defined as frail for the purpose of this analysis. The Acute Physiology and Chronic Health Evaluation (APACHE) III-J scoring system was used as a marker for illness severity. As the original APACHE III-J score utilises temperature as a variable, an adjusted score without the temperature criteria was used for this analysis. Age and chronic health components were retained in the modified APACHE III-J score and not included as separate covariates in the analysis. Multivariable analyses were performed using mixed effects logistic regression for mortality outcomes. To test the robustness of findings, a sensitivity analysis was undertaken using a different risk adjustment strategy including Sequential Organ Failure Assessment 2 score with available comorbidity data included as additional variables. Full models and handling of missing data is available in the Supplementary Material.

## Results

**Fig 1:**
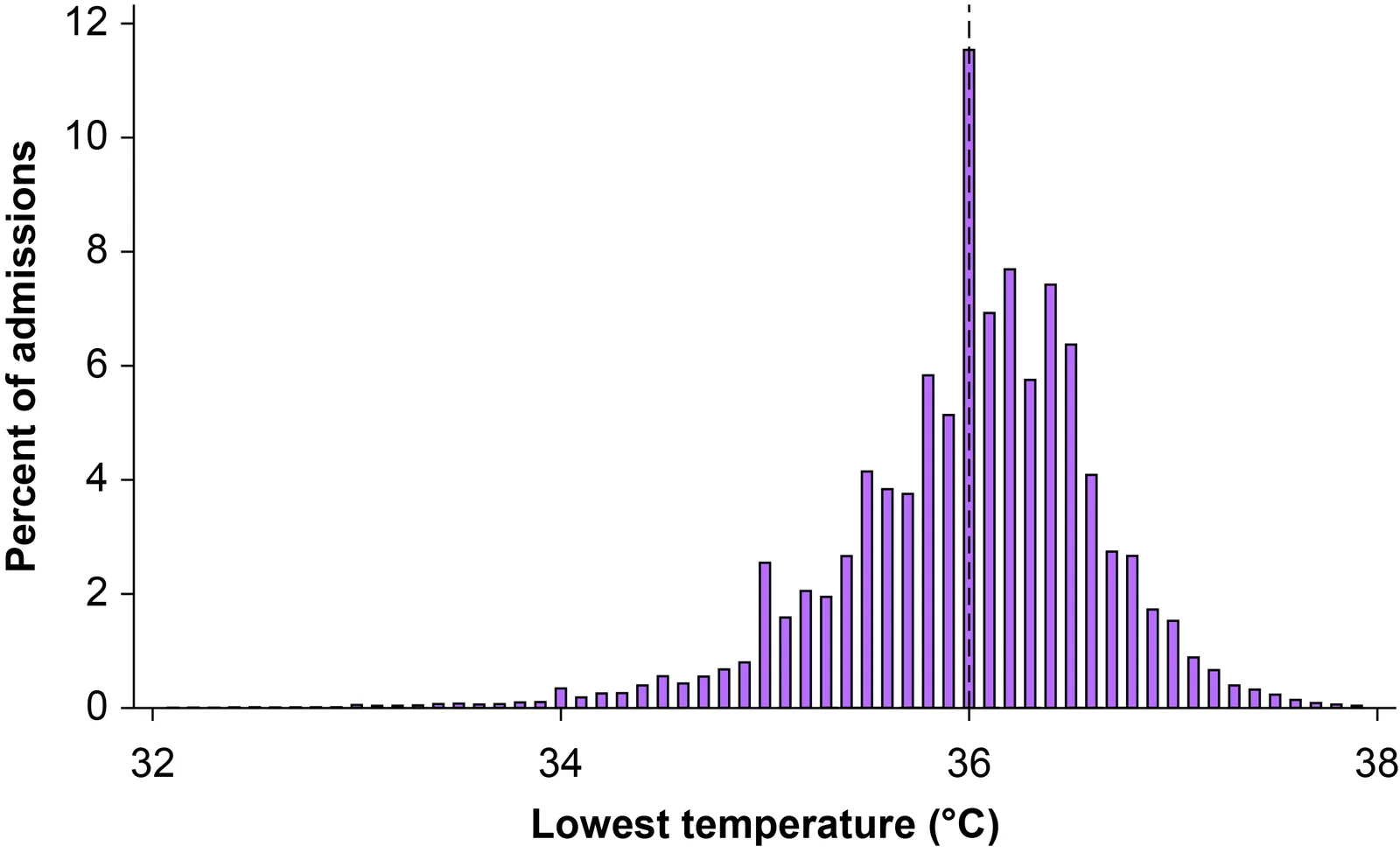
Histogram of lowest temperature in first 24 h in ICU (°C). Outliers <32°C not shown.

A total of 425 648 patients were admitted to ICU after surgery who met inclusion and exclusion criteria during the study period. Of the patients included in this study 260 537 (61.2%) were within normothermia range and 165 111 (38.8%) were hypothermic (Table 1 and Fig. 1).

**Table 1:** Demographics and baseline clinical characteristics of the normothermia 36–37.9°C and hypothermia <36°C groups. Numbers are count (percent) and median [interquartile range]. Mean (sd) is also reported for SOFA due to identical median and interquartile range. APACHE, Acute Physiology and Chronic Health Evaluation; LOS, length of stay; sd, standard deviation; SOFA, Sequential Organ Failure Assessment. ∗Highest SOFA/SOFA2 within first 24 h of ICU admission. Additional detail about each surgical group and the remaining patients classified as ‘other’ can be found in the supplementary material.

*n*	All patients	Normothermia	Hypothermia
	425 648	260 537 (61.2%)	165 111 (38.8%)
**Demographics**			
Age (yr)	66 [53–76]	65 [51–74]	69 [56–77]
Female sex	202 036 (47.5%)	124 668 (47.9%)	77 368 (46.9%)
Male sex	223 223 (52.5%)	135 663 (52.1%)	87 560 (53.1%)
Other	253 (0.1%)	135 (0.1%)	118 (0.1%)
**Clinical characteristics**			
SOFA score∗	2 [1–4]	2 [1–4]	2 [1–4]
SOFA2 score∗	2 [1–4]	2 [1–3]	2 [1–4]
APACHE III-J score	41 [31–53]	38 [28–50]	45 [35–58]
APACHE III-J score (temp adjusted)	40 [30–52]	38 [28–50]	43 [33–54]
Pre-ICU LOS (h)	10.4 (6.6–26.5)	10.6 (6.8–26.4)	10.2 [6.3–26.5]
Mechanical Ventilation within first 24 h	77 331 (18.2%)	39 785 (15.3%)	37 546 (22.7%)
Treatment limitation	15 426 (3.6%)	8 562 (3.3%)	6 864 (4.2%)
**Frailty**			
Yes	48 518 (11.4%)	27 602 (10.6%)	20 916 (12.7%)
No	207 248 (63.5%)	168 428 (64.6%)	101 820 (61.7%)
Unknown	106 882 (25.1%)	64 507 (24.8%)	42 375 (25.7%)
**Temperature first 24 h**			
Highest (°C)	37.0 [36.6–37.4]	37.1 [36.8–37.5]	36.7 [ 36.4–37.1]
Lowest (°C)	36.0 [35.7–36.4]	36.3 [36.1–36.6]	35.5 [35.2–35.8]
**Surgical type**			
Elective	311 533 (72.9%)	191 182 (73.1%)	120 351 (72.6%)
Vascular	37 855 (8.9%)	19 401 (7.4%)	18 454 (11.2%)
Thoracic	48 362 (11.4%)	29 195 (11.2%)	19 167 (11.6%)
Gastrointestinal	130 733 (30.7%)	83 237 (31.9%)	47 496 (28.8%)
Neurological	78 257 (18.4%)	48 573 (18.6%)	29 684 (18.0%)
Trauma	15 850 (3.7%)	9 685 (3.7%)	6 165 (3.7%)
Genitourinary	25 060 (5.9%)	15 485 (5.9%)	9 575 (5.8%)
Obstetric/Gynaecological	14 644 (3.4%)	9 714 (3.7%)	4 930 (3.0%)
Plastics and Orthopaedics	64 468 (15.1%)	38 359 (14.7%)	26 109 (15.8%)
Endocrine	8 482 (2.0%)	5 686 (2.2%)	2 796 (1.7%)
Other	1 937 (0.5%)	1 202 (0.5%)	735 (0.4%)
**Temperature groups (lowest temperature °C in first 24 h)**			
37–37.9	18 577 (4.4%)		
36–36.9	241 960 (56.8%)		
35–35.9	142 398 (33.5%)		
34–34.9	18 909 (4.4%)		
33–33.9	2 809 (0.7%)		
32–32.9	458 (0.1%)		
<32	537 (0.1%)

Patients who experienced postoperative hypothermia were more likely to be older, male and with higher illness severity scores (Table 1). Most admitted patients arrived as planned ICU admissions after elective operations and gastrointestinal surgery was the most common type of operation performed. The group with hypothermia had a higher proportion of patients who had undergone surgery categorised as, vascular and plastics/orthopaedics. All other operation types had a majority of patients maintain normothermia in the initial postoperative ICU period.

When stratified into groups based on their lowest temperature within 24 h of ICU admission, most patients fell within the 36–36.9°C group (56.8%) (Table 1). Patients with a lowest recorded temperature of <35°C accounted for just 5.3% of the total number of postoperative admissions.

Hospital mortality rates were higher in postoperative ICU patients with hypothermia compared with normothermia (Table 2). Mortality in the normothermic group was 2.1% compared with 4.3% in the hypothermic group (OR 2.05; 95% CI 1.97–2.01). The association persisted after adjusting for potential confounding factors (OR 1.34; 95% CI 1.28–1.39) (Table 3).

**Table 2:** Outcomes of patients with normothermia and hypothermia in the postoperative period. Numbers are count (percent) and median [interquartile range]. Adjusted for: hypothermia, sex, APACHE III-J Score (without temperature component), frailty, treatment limitation, elective surgery, operative group, invasive ventilation in first 24 h, pre-ICU hours. APACHE, Acute Physiology and Chronic Health Evaluation; CI, confidence interval; LOS, length of stay.

*n*	All Patients	Normothermia	Hypothermia	*P* value
	425 648	260 537 (61.2%)	165 111 (38.8%)	
**Primary outcome**				
Hospital mortality	12 528 (3.0%)	5 525 (2.1%, 95% CI 2.07%–2.18%)	7 003 (4.3% 95% CI 4.15%–4.25%)	<0.001
Unadjusted odds ratio		Reference group	2.05 (95% CI 1.97–2.12)	<0.001
Adjusted odds ratio		Reference group	1.34 (1.28–1.39)	<0.001
**Secondary outcomes**				
ICU mortality	6 438 (1.5%)	2 462 (0.9%)	3976 (2.4%)	<0.001
ICU readmission	17 380 (4.1%)	10 420 (4.0%)	6 960 (4.2%)	0.003
ICU LOS (days)	1.0 [0.8–2.1]	1.0 [0.8–2.0]	1.1 [ 0.9–2.3]	<0.001
Hospital LOS (days)	5.9 [ 3.1–10.9]	5.9 [3.0–10.7]	6.0 [3.4–11.2]	<0.001

**Table 3:** Hospital mortality by temperature group, 37–37.9°C reference group. Adjusted for: hypothermia, sex, APACHE III Score (without temperature component), frailty, and operative group. APACHE, Acute Physiology and Chronic Health Evaluation; CI, confidence interval; OR, odds ratio.

Lowest temperature °C in first 24 h	Hospital mortality, *n* (%, 95% CI)	OR (95% CI)	*P* value	OR (95% CI) adjusted	*P* value adjusted
37–37.9	649 (3.5%, 3.2%–3.8%)	1 [reference]	-	-	-
36–36.9	4 876 (2.0%, 2.0%–2.1%)	0.57 (0.53–0.63)	<0.001	0.87 (0.79–0.96)	0.004
35–35.9	4 612(3.2%, 3.2%–3.3%)	0.93 (0.85–1.00)	0.07	1.04 (0.95–1.14)	0.41
34–34.9	1 653 (8.8%, 8.4%–9.2%)	2.65 (2.41–2.91)	<0.001	1.62 (1.45–1.80)	<0.001
33–33.9	524 (18.7%, 17.2%–20.2%)	6.34 (5.60–7.17)	<0.001	2.13 (1.82–2.46)	<0.001
32–32.9	138 (30.2%, 26.0%–34.6%)	11.92 (9.61–14.78)	<0.001	3.04 (2.30–4.00)	<0.001
<32	76 (14.2%, 11.3%–17.4%)	4.54 (3.52–5.86)	<0.001	1.63 (1.19–2.23)	0.002

**Fig 2:**
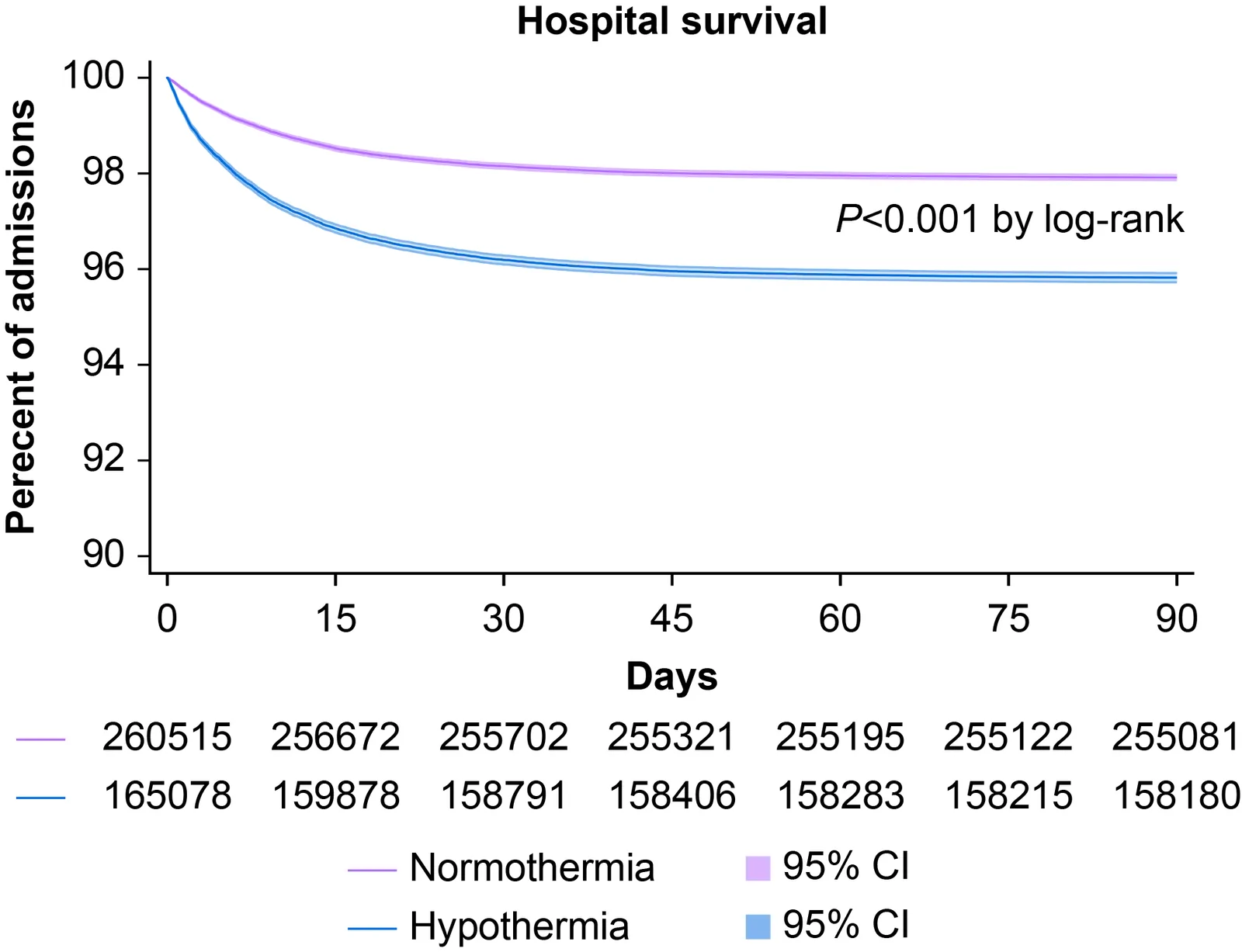
Kaplan–Meier curve displaying patient survival in hospital to 90 days comparing patients with normothermia and those with hypothermia. CI, confidence interval.

**Fig 3:**
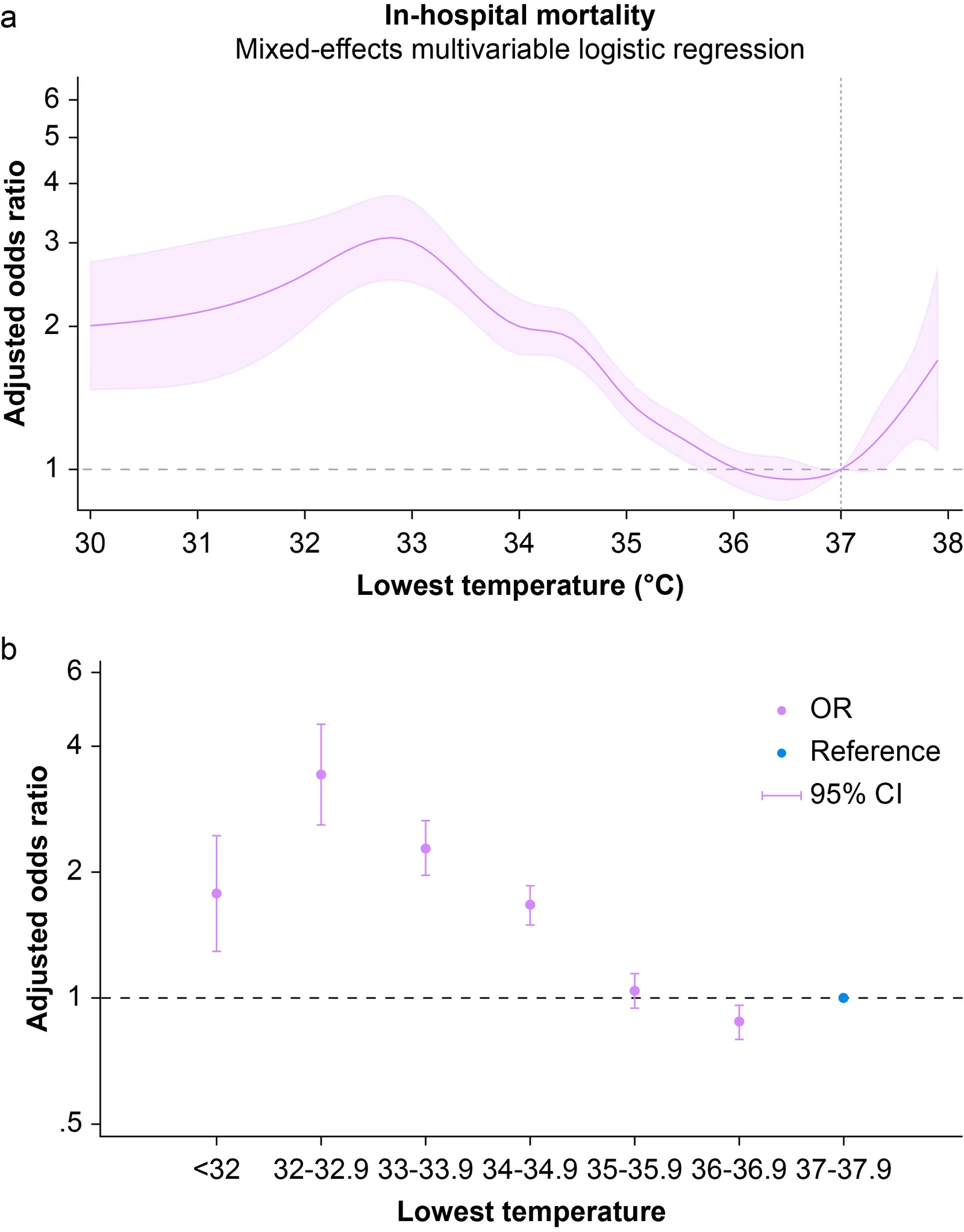
Adjusted in-hospital mortality rates comparing patients' lowest recorded temperature within 24 h of ICU admission divided into 1°C temperature categories. 37–37.9°C is used as reference. Data is displayed as both (a) spline curve analysis and (b) ORs demonstrating increasing OR of mortality outcome with lower temperatures. Adjusted for: hypothermia, sex, APACHE III-J score (without temperature component), frailty, treatment limitation, elective surgery, operative group, invasive ventilation in first 24 h, and pre-ICU hours. APACHE, Acute Physiology and Chronic Health Evaluation; CI, confidence interval; OR, odds ratio.

When divided into 1°C temperature categories, patients with lower temperature experienced much higher unadjusted odds of mortality, with peak mortality rate in the 32–32.9°C group (OR 11.92; 95% CI 9.61–14.78). After adjusting for confounders, mortality remained highest in this group (OR 3.03; 95% CI 2.30–4.00; Table 3). The lowest adjusted odds of mortality were observed in the 36–36.9°C band of the normothermic group and the odds of mortality did not increase above the 37–37.9°C reference group until temperature dropped below 35°C. The survival curve is displayed in Fig. 2 and mortality trends between temperature groups are displayed in Fig. 3.

In the secondary outcome analysis, patients with hypothermia had longer ICU stay and longer hospital stay compared with the normothermia group (Table 3). Overall, the median ICU length of stay was just over one day, and the median hospital length of stay was approximately 6 days in both groups. There was a small increase in readmission rates for the hypothermic group, with 4.2% of hypothermic patients requiring ICU readmission compared with 4.0% of normothermic patients (*P* =<.001). Mortality during the ICU stay was also higher in the hypothermic group, with a mortality rate of 2.4% compared with 0.9% in the normothermia group (*P*<0.001).

## Discussion

### Key findings

In this large volume retrospective cohort study, hypothermia was a common occurrence in noncardiac surgical postoperative ICU patients, occurring in 38.8% of admitted patients. Hypothermia within 24 h of ICU admission in this patient cohort was associated with an increased in hospital mortality rate. This finding persisted when adjusted for illness severity (including age), sex, procedure type and frailty in multi-variate analysis and occurred as both increased in-hospital and ICU mortality. When stratifying patients, in hospital mortality rates increased with lower temperature categories.

Additional patient outcomes between normothermic and hypothermic groups showed small but statistically significant differences in both ICU and hospital lengths of stay. There was an overall slight increase in ICU and hospital length of stay in unadjusted analysis. Readmission rates to ICU were similar between the two groups.

### Relationship to previous findings

The incidence of hypothermia in this study at 38.8% is similar to that demonstrated in earlier research. One study of postoperative ICU patients reported a hypothermia rate of 57.1%.^17^ Another retrospective observational study from 2013 showed an incidence of 46%, and a 2009 study reported an incidence of 35%.^1^

Our findings of increased mortality in postoperative patients with hypothermia are consistent with some previous smaller studies. A single centre analysis of 5,968 patients in a South Korean surgical ICU found that deviation from normothermia was associated with increased 1 yr mortality, however this study only included a single temperature measurement within 10 min of ICU admission.^18^ The 2009 study cited earlier also demonstrated increased mortality although its included cardiac surgery patients, which were excluded from our criteria.^19^ The subsequent 2013 study of 50 689 elective noncardiac surgery admissions did not demonstrate a mortality difference between groups in contrast to the findings of our own study which included both elective and emergency surgical patients.^1^

The above studies looked at the perioperative cohort, but the body of evidence related to intraoperative temperature management is also of value in our analysis. Aggressive warming intraoperatively has been investigated as a possible therapeutic model to reduce major cardiovascular events in patient undergoing noncardiac surgery. A large-scale randomised trial found that aggressive warming to a temperature of 37°C did not improve outcomes compared with routine temperature target management of 35.5°C.^20^ This is consistent with our findings which demonstrated mortality increasing with lowest temperature below 35°C and being comparable between the 35–35.9°C and 37–37.9°C temperature groups.

### Implications

Hypothermia is still highly prevalent and may result in significant harm to postoperative patients in the ICU. The lack of change in its incidence over the years suggests that the impact of hypothermia on mortality may be under-recognised. Routine warming and aims of normothermia in the postoperative intensive care patients may be beneficial as routine practise until further study can confirm a causative link between hypothermia and adverse outcomes in the intensive care population. Interventional studies utilising active warming in ICU would be beneficial in confirming these adverse effects. This data suggests a temperature range of 36–36.9°C may be associated with the lowest risk of mortality in this patient cohort.

### Strengths and limitations

A major strength of our study is the large volume of patients, 425,648 included in the studied cohort. Regarding generalisability of our results, this patient cohort included admissions across most intensive care sites around Australia and New Zealand and was taken over a contemporary time period. Our findings were consistent after adjustment for confounding factors of illness severity and are in keeping with previous studies.

This study had a number of limitations. First is the retrospective nature of the study and reliance of routine observations documented by multiple hospital sites. There may be inaccuracies in the recording of data or missing values in the registry. Certain categories in our registry such as clinical frailty had a high percentage of missing data which may impact its inclusion in our multivariable analysis. Potential confounders may be the existence of other variables not captured in the dataset such as duration of surgery, intraoperative warming techniques or ASA physical status classification. Next, the data set utilised recorded lower temperature measurement within the first 24 h of admission but did not indicate the duration of hypothermia, or whether patients were hypothermic or hyperthermic at the time of ICU admission or later in their postoperative course. It was also not possible to separate intentional hypothermic management from inadvertent hypothermia.

Finally, the method of temperature measurement may have varied between sites and inaccuracies may exist between different modalities of measurement. Our database has the recorded lowest and highest core temperature with sites including oral, tympanic, nasopharyngeal, rectal, oesophageal, pulmonary artery and bladder. The exact site is not specified and there is an option for skin or axillary thermometer measurements if a core temperature was unable to be obtained. This may lead to inaccuracies between patient measurements and lead to measurement bias within our results.

### Conclusions

We report that hypothermia with temperature <36°C in non cardiac postoperative ICU patients to be a common occurrence and associated with increased in hospital mortality. Mortality rates increased alongside severity of hypothermia and this trend continued until the final category of patients with temperature <32°C.

Hypothermia may be a modifiable risk factor in postoperative ICU patients and could be the target for further intervention to improve patient survival.

## Authors’ contributions

Study design, data access, manuscript drafting, critical review of manuscript: JW

Study design, data access, data analysis and interpretation, critical review of manuscript: MD

Study design, data access, critical review of manuscript: TH

Study design, data access, critical review of manuscript: TE

Study design, data access, data analysis and interpretation, critical review of manuscript: DP

## Declaration of interests

The author declares that they have no conflict of interest.

## References

[1] Karalapillai D., Story D., Hart G.K. (2013). Postoperative hypothermia and patient outcomes after major elective non-cardiac surgery. Anaesthesia.

[2] Abelha F.J., Castro M.A., Neves A.M., Landeiro N.M., Santos C.C. (2005). Hypothermia in a surgical intensive care unit. BMC Anesthesiol.

[3] Sessler D.I. (2000). Perioperative heat balance. Anesthesiology.

[4] Sessler D.I. (2013). The thermoregulation story. Anesthesiology.

[5] Campbell G., Alderson P., Smith A.F., Warttig S. (2015). Warming of intravenous and irrigation fluids for preventing inadvertent perioperative hypothermia. Cochrane Database Syst Rev.

[6] National Institute for Health and Care Excellence [guidelines] (2019). https://www.ncbi.nlm.nih.gov/books/NBK554181/.

[7] Kurz A., Sessler D.I., Lenhardt R. (1996). Perioperative normothermia to reduce the incidence of surgical-wound infection and shorten hospitalization. Study of Wound Infection and Temperature Group. N Engl J Med.

[8] Reynolds L., Beckmann J., Kurz A. (2008). Perioperative complications of hypothermia. Best Pract Res Clin Anaesthesiol.

[9] Schmied H., Kurz A., Sessler D.I., Kozek S., Reiter A. (1996). Mild hypothermia increases blood loss and transfusion requirements during total hip arthroplasty. Lancet.

[10] Rauch S., Miller C., Bräuer A., Wallner B., Bock M., Paal P. (2021). Perioperative hypothermia-a narrative review. Int J Environ Res Public Health.

[11] Buggy D.J., Crossley A.W. (2000). Thermoregulation, mild perioperative hypothermia and postanaesthetic shivering. Br J Anaesth.

[12] Riley C., Andrzejowski J. (2018). Inadvertent perioperative hypothermia. BJA Educ.

[13] Lenhardt R., Marker E., Goll V. (1997). Mild intraoperative hypothermia prolongs postanesthetic recovery. Anesthesiology.

[14] Ralph N., Gow J., Conway A. (2020). Costs of inadvertent perioperative hypothermia in Australia: a cost-of-illness study. Collegian.

[15] Secombe P., Millar J., Litton E. (2023). Thirty years of ANZICS CORE: a clinical quality success story. Crit Care Resusc.

[16] Subramaniam A., Ueno R., Tiruvoipati R., Srikanth V., Bailey M., Pilcher D. (2022). Comparison of the predictive ability of clinical frailty scale and hospital frailty risk score to determine long-term survival in critically ill patients: a multicentre retrospective cohort study. Crit Care.

[17] Kongsayreepong S., Chaibundit C., Chadpaibool J. (2003). Predictor of core hypothermia and the surgical intensive care unit. Anesth Analg.

[18] Kim J., Oh T.K., Lee J., Kim S., Song I.A. (2019). Association of immediate postoperative temperature in the surgical intensive care unit with 1-year mortality: retrospective analysis using digital axillary thermometers. Acute Crit Care.

[19] Karalapillai D., Story D.A., Calzavacca P., Licari E., Liu Y.L., Hart G.K. (2009). Inadvertent hypothermia and mortality in postoperative intensive care patients: retrospective audit of 5050 patients. Anaesthesia.

[20] Sessler D.I., Pei L., Li K. (2022). Aggressive intraoperative warming versus routine thermal management during non-cardiac surgery (PROTECT): a multicentre, parallel group, superiority trial. Lancet.

