# Supplementary material for: Hypothermia in the intensive care unit after surgery: a registry based retrospective cohort study
Source: BJA Open. 2026 Aug 13;19:100556. doi: 10.1016/j.bjao.2026.100556 (PMC13499147; doi:10.1016/j.bjao.2026.100556)
Supplement: Supplementary file 1 [file mmc1.docx]

**Supplementary Appendix**

**Hypothermia in the intensive care unit following surgery: a registry based retrospective cohort study**

Dr Jeremy Weiss, Dr Matthew Durie, Dr Tamishta Hensman, Dr Tess Evans, Dr David Pilcher

**Contents:**

1. Study inclusions and exclusions flowchart
2. Missing data
3. Procedure group diagnoses (including ‘other’)
4. Hospital mortality: multivariable models
   a. Hypothermia vs. normothermia
   b. 1-degree temperature increments
5. ICU mortality: multivariable models
   a. Hypothermia vs. normothermia
   b. 1-degree temperature increments
6. Figure S1: 90-day hospital survival curve by 1-degree increments
7. Figure S2: Unadjusted hospital mortality vs temperature (spline)
8. Figure S3: Hospital mortality vs. temperature (adjusted with SOFA2)
9. Participating hospital sites

**1. Study inclusions and exclusions flowchart**

Patients admitted to participating ICUs Jan 2018 - Jun 2023

Exclusions: -Age < 18 = 6,113 -Non-surgical = 485,193 -Cardiac surgical = 116,699 -Palliative admissions = 1,376 -ICU readmission episode = 13,966 -Temperature missing = 5,737 -Lowest temp in 24 hours >38°C = 349 -

Final study population

Normothermia

Hypothermia

**2. Missing Data**

|  | All (425,648) | Normothermia (260,537) | Hypothermia (165,111 | Handling |
| --- | --- | --- | --- | --- |
| Age | 0 | 0 | 0 | NA |
| Sex | 136 (0.03) | 71 (0.03) | 65 (0.04) | Excl |
| APACHE III-j score | 0 | 0 | 0 | NA |
| APACHE comorbidity | 63 (0.01) | 41 (0.02) | 22 (0.01) | Excl |
| SOFA2 domain |  |  |  |  |
| Haem | 39,130(9.2) | 24,122 (9.3) | 15,008 (9.1) | Min |
| Resp | 0 | 0 | 0 | N/A |
| Renal | 17,778(4.2) | 11,500 (4.4) | 6,278 (3.8) | Min |
| Cardiovascular | 650(0.15) | 406 (0.16) | 244 (0.15) | Min |
| Liver | 58,804 (13.8) | 36,531 (14.0) | 22,273 (13.5) | Min |
| Neurological | 10,080 (2.4) | 5,915 (2.3) | 4,140 (2.5) | Min |
| Ventilated on day 1 ICU | 252 (0.1) | 142 (0.1) | 110 (0.1) | Min |
| Frailty | 106,882 (25.1) | 64,507 (24.7) | 42,375 (25.6) | Cat |
| Treatment limitation | 0 | 0 | N/A | N/A |
| Pre ICU hours | 129 (0.03) | 67 (0.03) | 62 (0.03) | Cat |
| Diabetes | 88,252 (19.9) | 50,020 (19.2) | 34,531 (20.9) | No |
| Elective surgery | 510 (0.12) | 350 (0.13) | 160 (0.10) | Cat |
| Surgical type | 1,739 (0.04) | 1,062 (0.04) | 634 (0.04) | Cat |
| *Outcome* | | | | |
| ICU mortality | 1,172 (0.3) | 474 (0.3) | 698 (0.3) | Excl/Cen |
| Hospital mortality | 1,158(0.3) | 660 (0.3) | 498 (0.3) | Excl/Cen |
| ICU LOS | 1,078 (0.3) | 641 (0.2) | 437 (0.3) | Cen |
| Hospital LOS | 1,469 (0.3) | 852 (0.3) | 617 (0.4) | Cen |
|  |  |  |  |  |

Min: infer score component as minimum (zero) or absent. No: infer as not present. Cat: include in multivariable analysis as separate category. Excl: excluded from analysis. Cen: censored at 90 days as alive/still admitted.

**3. Full list of procedural diagnoses**

| **Procedure group** | **Subgroup** | **N** | **%** |
| --- | --- | --- | --- |
| Gastrointestinal | GI perforation/rupture | 8,480 | 6.5 |
|  | GI bleeding | 7,640 | 5.8 |
|  | GI obstruction | 12,336 | 9.4 |
|  | GI neoplasm | 38,360 | 29.3 |
|  | Cholecystitis/Cholangitis | 8,639 | 6.6 |
|  | Liver transplant | 1,373 | 1.1 |
|  | Other GI diseases | 45,140 | 34.5 |
|  | Fistula/Abscess surgery | 1,937 | 1.5 |
|  | GI vascular ischaemia resection surgery | 2,086 | 1.6 |
|  | Pancreatitis | 620 | 0.5 |
|  | Peritonitis | 1,390 | 1.1 |
|  | Other GI inflammatory disease | 2,732 | 2.1 |
| Neurological |  |  |  |
|  | Intracerebral haemorrhage | 2,039 | 2.6 |
|  | Subdural/Epidural haematoma | 2,709 | 3.5 |
|  | Subarachnoid haemorrhage | 4,183 | 5.4 |
|  | Laminectomy/Spinal cord surgery | 35,773 | 45.7 |
|  | Craniotomy for neoplasm | 15,758 | 20.1 |
|  | Other neurologic disease | 17,795 | 22.7 |
| Plastic & Orthopaedic |  |  |  |
|  | Orthopaedic surgery | 52,447 | 81.4 |
|  | Plastic surgery | 7,276 | 11.3 |
|  | Cellulitis/soft tissue infection | 4,743 | 7.4 |
| Thoracic |  |  |  |
|  | Respiratory infection | 3,284 | 6.8 |
|  | Respiratory neoplasm – lung | 14,084 | 29.1 |
|  | Respiratory neoplasm – other | 7,472 | 15.5 |
|  | Other respiratory diseases | 23,522 | 48.6 |
| Vascular |  |  |  |
|  | Peripheral vascular disease | 4,921 | 13.0 |
|  | Peripheral artery bypass graft | 3,063 | 8.1 |
|  | Elective AAA | 4,879 | 12.9 |
|  | Carotid endarterectomy | 7,084 | 18.7 |
|  | Other cardiovascular diseases | 10,218 | 27.0 |
|  | Dissecting aortic aneurysm | 1,775 | 4.7 |
|  | Ruptured aortic aneurysm | 1,009 | 2.7 |
|  | Aorto-femoral bypass graft | 1,080 | 2.9 |
|  | Endoluminal aortic repair | 3,826 | 10.1 |
| Genitourinary |  |  |  |
|  | Renal neoplasm | 10,082 | 40.2 |
|  | Other renal diseases | 7,452 | 29.7 |
|  | Kidney transplant | 1,215 | 4.9 |
|  | Genitourinary surgery/procedure | 6,311 | 25.2 |
| Trauma |  |  |  |
|  | Head trauma +/- multi trauma | 3,572 | 22.5 |
|  | Multiple trauma excluding head | 10,348 | 65.3 |
|  | Burns | 397 | 2.5 |
|  | Multi trauma with spinal injury | 959 | 6.1 |
|  | Isolated cervical spine injury | 574 | 3.6 |
| Obstetric & Gynaecology |  |  |  |
|  | Hysterectomy | 5,126 | 35.0 |
|  | Pregnancy-related disorder | 6,850 | 46.8 |
|  | Other gynaecological disease | 2,668 | 18.2 |
| Endocrine |  |  |  |
|  | Adrenalectomy | 1,135 | 13.4 |
|  | Thyroid and parathyroid surgery | 6,243 | 73.6 |
|  | Metabolic/endocrine surgery, other | 630 | 7.4 |
| Other |  |  |  |
|  | Rhythm disturbance | 1 | 0.05 |
|  | Myocardial infarct | 1 | 0.05 |
|  | Aspiration pneumonia | 1 | 0.05 |
|  | Emphysema | 2 | 0.10 |
|  | Asthma | 1 | 0.05 |
|  | Sleep apnoea | 4 | 0.21 |
|  | Hanging | 1 | 0.05 |
|  | Acid base disturbance | 3 | 0.15 |
|  | Haematological disease | 184 | 9.50 |
|  | Missing | 1,739 | 89.8 |

**4a.i Hospital mortality multivariable regression model: hypothermia vs. normothermia (APACHE III-J)**

|  | OR | 95% CI | P-value |
| --- | --- | --- | --- |
| Hypothermia | 1.336 | [1.2820,1.3915] | <0.001 |
| Gender |  |  |  |
| Male | 1.105 | [1.0598,1.1515] | <0.001 |
| Other | 1.053 | [0.3496,3.1735] | 0.93 |
| APACHE III-J (no temperature) | 1.051 | [1.0498,1.0519] | <0.001 |
| Ventilation on ICU day 1 | 2.791 | [2.6653,2.9236] | <0.001 |
| Frailty Score |  |  |  |
| 2 | 1.069 | [0.8987,1.2720] | 0.45 |
| 3 | 1.227 | [1.0376,1.4499] | 0.02 |
| 4 | 1.686 | [1.4246,1.9955] | <0.001 |
| 5 | 2.037 | [1.7124,2.4225] | <0.001 |
| 6 | 2.481 | [2.0850,2.9516] | <0.001 |
| 7 | 3.186 | [2.6456,3.8357] | <0.001 |
| 8 | 5.742 | [4.4543,7.4007] | <0.001 |
| Missing | 1.713 | [1.4527,2.0188] | <0.001 |
| Treatment limitation | 2.818 | [2.6619,2.9835] | <0.001 |
| Elective Surgery |  |  |  |
| Yes | 0.458 | [0.4365,0.4814] | <0.001 |
| Unknown | 0.923 | [0.6089,1.3976] | 0.70 |
| Operative group |  |  |  |
| Gastrointestinal | (ref) |  |  |
| Neurological | 2.166 | [2.0430,2.2968] | <0.001 |
| Plastics & Ortho. | 0.757 | [0.7035,0.8152] | 0.003 |
| Thoracic | 1.095 | [1.0008,1.1985] | 0.05 |
| Vascular | 1.379 | [1.2879,1.4771] | <0.001 |
| Genitourinary | 0.602 | [0.5394,0.6711] | <0.001 |
| Trauma | 1.429 | [1.3272,1.5376] | <0.001 |
| Obst/Gyn | 0.320 | [0.2385,0.4283] | <0.001 |
| Endocrine | 0.386 | [0.2597,0.5741] | <0.001 |
| Other | 1.511 | [1.1824,1.9300] | 0.001 |
| Pre-ICU hours | 1.000 | [1.0000,1.0000] | 0.02 |
| Intercept | 0.001 | [0.0007,0.0010] | <0.001 |
| *Number of observations* | *424,230* |  |  |

**4a.ii Hospital mortality multivariable regression model: hypothermia vs. normothermia (SOFA2)**

|  | OR | 95% CI | P-value |
| --- | --- | --- | --- |
| Hypothermia | 1.390 | [1.3298,1.4531] | <0.001 |
| Age | 1.025 | [1.0234,1.0268] | <0.001 |
| Gender |  |  |  |
| Male | 1.016 | 1.016 | 0.48 |
| Other | 0.333 | [0.0424,2.6147] | 0.30 |
| SOFA2 | 1.342 | [1.3315,1.3524] | <0.001 |
| Ventilation on ICU day 1 | 2.969 | [2.8202,3.1265] | <0.001 |
| Frailty Score |  |  |  |
| 2 | 0.945 | [0.7836,1.1398] | 0.55 |
| 3 | 0.993 | [0.8276,1.1909] | 0.94 |
| 4 | 1.368 | [1.1381,1.6448] | 0.001 |
| 5 | 1.647 | [1.3616,1.9929] | <0.001 |
| 6 | 2.051 | [1.6930,2.4835] | <0.001 |
| 7 | 2.639 | [2.1502,3.2400] | <0.001 |
| 8 | 4.752 | [3.5808,6.3053] | <0.001 |
| Missing | 1.527 | [1.2769,1.8259] | <0.001 |
| Treatment limitation | 2.891 | [2.7131,3.0802] | <0.001 |
| Elective Surgery |  |  |  |
| Yes | 0.374 | [0.3550,0.3943] | <0.001 |
| Unknown | 0.926 | [0.5559,1.5439] | 0.77 |
| Operative group |  |  |  |
| Gastrointestinal | (ref) |  |  |
| Neurological | 2.320 | [2.1785,2.4704] | <0.001 |
| Plastics & Ortho. | 0.810 | [0.7468,0.8793] | <0.001 |
| Thoracic | 1.032 | [0.9354,1.1394] | 0.53 |
| Vascular | 1.122 | [1.0403,1.2107] | 0.003 |
| Genitourinary | 0.537 | [0.4755,0.6060] | <0.001 |
| Trauma | 1.425 | [1.3171,1.5419] | <0.001 |
| Obst/Gyn | 0.336 | [0.2442,0.4636] | <0.001 |
| Endocrine | 0.446 | [0.2931,0.6791] | <0.001 |
| Other | 1.638 | [1.2259,2.1895] | 0.001 |
|  |  |  |  |
| Pre-ICU hours | 1.000 | [1.0000,1.0000] | 0.005 |
| Chronic Respiratory | 1.270 | [1.1691,1.3786] | <0.001 |
| Chronic Cardiovascular | 0.886 | [0.8183,0.9604] | 0.003 |
| Chronic Liver | 0.870 | [0.7766,0.9757] | 0.02 |
| Chronic Renal | 0.774 | [0.6990,0.8576] | <0.001 |
| Malignancy | 2.008 | [1.8577,2.1712] | <0.001 |
| Immunosuppressed | 1.206 | [1.1101,1.3099] | <0.001 |
| Diabetes | 1.075 | [1.0185,1.1342] | 0.009 |
| Intercept | 0.001 | [0.0007,0.0011] | <0.001 |
| *Number of observations* | *334,757* |  |  |

**4b.i Hospital mortality multivariable regression model: 1-degree increments (APACHE III-J)**

|  | OR | 95% CI | P-value |
| --- | --- | --- | --- |
| Lowest temperature (°C) |  |  |  |
| 36-36.9 | 0.870 | [0.7918,0.9563] | 0.004 |
| 35-35.9 | 1.041 | [0.9466,1.1448] | 0.41 |
| 34-34.9 | 1.621 | [1.4545,1.8060] | <0.001 |
| 33-33.9 | 2.125 | [1.8295,2.4686] | <0.001 |
| 32-32.9 | 3.034 | [2.2995,4.0026] | <0.001 |
| <32 | 1.634 | [1.1922,2.2397] | 0.002 |
| Gender |  |  |  |
| Male | 1.115 | [1.0691,1.1620] | <0.001 |
| Other | 1.023 | [0.3360,3.1172] | 0.97 |
| APACHE III-J (no temperature) | 1.050 | [1.0491,1.0512] | <0.001 |
| Ventilation on ICU day 1 | 2.659 | [2.5370,2.7860] | <0.001 |
| Frailty Score |  |  |  |
| 2 | 1.073 | [0.9014,1.2783] | 0.43 |
| 3 | 1.241 | [1.0493,1.4688] | 0.01 |
| 4 | 1.718 | [1.4499,2.0346] | <0.001 |
| 5 | 2.090 | [1.7555,2.4878] | <0.001 |
| 6 | 2.545 | [2.1373,3.0309] | <0.001 |
| 7 | 3.277 | [2.7194,3.9494] | <0.001 |
| 8 | 5.773 | [4.4757,7.4462] | <0.001 |
| Missing | 1.742 | [1.4767,2.0558] | <0.001 |
| Treatment Limitation | 2.834 | [2.6763,3.0006] | <0.001 |
| Elective Surgery |  |  |  |
| Yes | 0.459 | [0.4374,0.4825] | <0.001 |
| Unknown | 0.905 | [0.5969,1.3717] | 0.64 |
| Operative group |  |  |  |
| Gastrointestinal | (ref) |  |  |
| Neurological | 2.122 | [2.0008,2.2502] | <0.001 |
| Plastics & Ortho. | 0.753 | [0.6995,0.8106] | <0.001 |
| Thoracic | 1.087 | [0.9933,1.1897] | 0.07 |
| Vascular | 1.311 | [1.2230,1.4046] | <0.001 |
| Genitourinary | 0.597 | [0.5351,0.6658] | <0.001 |
| Trauma | 1.388 | [1.2890,1.4946] | <0.001 |
| Obst/Gyn | 0.317 | [0.2367,0.4248] | <0.001 |
| Endocrine | 0.386 | [0.2594,0.5745] | <0.001 |
| Other | 1.491 | [1.1662,1.9067] | 0.001 |
| Pre ICU hours | 1.000 | [1.0000,1.0000] | 0.01 |
| Intercept | 0.001 | [0.0008,0.0012] | <0.001 |
| *Number of observations* | *424,230* |  |  |

**4b.ii Hospital mortality multivariable regression model: 1-degree increments (SOFA2)**

|  | OR | 95% CI | P-value |
| --- | --- | --- | --- |
| Lowest temperature (°C) |  |  |  |
| 36-36.9 | 0.924 | [0.8348,1.0232] | 0.13 |
| 35-35.9 | 1.143 | [1.0320,1.2667] | 0.01 |
| 34-34.9 | 1.802 | [1.6045,2.0245] | <0.001 |
| 33-33.9 | 2.316 | [1.9736,2.7167] | <0.001 |
| 32-32.9 | 2.800 | [2.0916,3.7488] | <0.001 |
| <32 | 1.571 | [1.1135,2.2157] | 0.01 |
| Age | 1.025 | [1.0233,1.0267] | <0.001 |
| Gender |  |  |  |
| Male | 1.027 | [0.9815,1.0743] | 0.25 |
| Other | 0.308 | [0.0380,2.4970] | 0.27 |
| SOFA2 | 1.335 | [1.3241,1.3451] | <0.001 |
| Ventilation on ICU day 1 | 2.843 | [2.6990,2.9953] | <0.001 |
| Frailty Score |  |  |  |
| 2 | 0.945 | [0.7828,1.1401] | 0.55 |
| 3 | 0.995 | [0.8291,1.1947] | 0.96 |
| 4 | 1.377 | [1.1447,1.6564] | 0.001 |
| 5 | 1.669 | [1.3786,2.0202] | <0.001 |
| 6 | 2.075 | [1.7124,2.5148] | <0.001 |
| 7 | 2.677 | [2.1801,3.2884] | <0.001 |
| 8 | 4.744 | [3.5736,6.2982] | <0.001 |
| Missing | 1.543 | [1.2896,1.8464] | <0.001 |
| Treatment limitation | 2.891 | [2.7131,3.0813] | <0.001 |
| Elective Surgery |  |  |  |
| Yes | 0.374 | [0.3553,0.3947] | <0.001 |
| Unknown | 0.918 | [0.5494,1.5336] | 0.74 |
| Operative group |  |  |  |
| Gastrointestinal | (ref) |  |  |
| Neurological | 2.276 | [2.1367,2.4238] | <0.001 |
| Plastics & Ortho. | 0.807 | [0.7433,0.8752] | <0.001 |
| Thoracic | 1.025 | [0.9287,1.1316] | 0.62 |
| Vascular | 1.065 | [0.9861,1.1497] | 0.11 |
| Genitourinary | 0.535 | [0.4738,0.6039] | <0.001 |
| Trauma | 1.392 | [1.2860,1.5066] | <0.001 |
| Obst/Gyn | 0.336 | [0.2441,0.4629] | <0.001 |
| Endocrine | 0.445 | [0.2923,0.6788] | <0.001 |
| Other | 1.614 | [1.2077,2.1577] | 0.001 |
| Pre-ICU hours | 1.000 | [1.0000,1.0000] | 0.003 |
| Chronic Respiratory | 1.281 | [1.1793,1.3907] | <0.001 |
| Chronic Cardiovascular | 0.892 | [0.8236,0.9668] | 0.005 |
| Chronic Liver | 0.886 | [0.7898,0.9934] | 0.04 |
| Chronic Renal | 0.790 | [0.7126,0.8747] | <0.001 |
| Malignancy | 2.008 | [1.8572,2.1709] | <0.001 |
| Immunosuppressed | 1.209 | [1.1133,1.3139] | <0.001 |
| Diabetes | 1.086 | [1.0288,1.1460] | 0.003 |
| Intercept | 0.001 | [0.0008,0.0013] | <0.001 |
| *Number of observations* | *334,757* |  |  |

**5a.i ICU mortality multivariable regression model: hypothermia vs. normothermia (APACHE III-j)**

|  | OR | 95% CI | P-value |
| --- | --- | --- | --- |
| Hypothermia | 1.455 | [1.3724,1.5419] | <0.001 |
| Gender |  |  |  |
| Male | 1.013 | [0.9553,1.0734] | 0.67 |
| Other | 0.515 | [0.0609,4.3596] | 0.54 |
| APACHE III-J (no temperature) | 1.054 | [1.0522,1.0549] | <0.001 |
| Ventilation on ICU day 1 | 5.735 | [5.3252,6.1766] |  |
| Frailty Score |  |  |  |
| 2 | 0.999 | [0.8151,1.2247] | 0.99 |
| 3 | 1.023 | [0.8402,1.2459] | 0.82 |
| 4 | 1.121 | [0.9168,1.3705] | 0.27 |
| 5 | 1.099 | [0.8895,1.3581] | 0.38 |
| 6 | 1.344 | [1.0878,1.6607] | 0.006 |
| 7 | 1.418 | [1.1208,1.7931] | 0.004 |
| 8 | 2.841 | [2.0334,3.9695] | <0.001 |
| Missing | 1.111 | [0.9159,1.3487] | 0.29 |
| Treatment Limitation | 2.839 | [2.6248,3.0697] | <0.001 |
| Elective Surgery |  |  |  |
| Yes | 0.372 | [0.3446,0.4018] | <0.001 |
| Unknown | 0.867 | [0.4809,1.5623] | 0.63 |
| Operative group |  |  |  |
| Gastrointestinal |  |  |  |
| Neurological | 2.966 | [2.7360,3.2148] | <0.001 |
| Plastics & Ortho. | 0.746 | [0.6620,0.8396] | <0.001 |
| Thoracic | 0.873 | [0.7467,1.0205] | 0.09 |
| Vascular | 2.087 | [1.9057,2.2850] | <0.001 |
| Genitourinary | 0.603 | [0.5073,0.7170] | <0.001 |
| Trauma | 2.018 | [1.8369,2.2179] | <0.001 |
| Obst/Gyn | 0.303 | [0.1919,0.4770] | <0.001 |
| Endocrine | 0.675 | [0.3825,1.1910] | 0.8 |
| Other | 2.280 | [1.6549,3.1420] | <0.001 |
| Pre ICU hours | 1.000 | [1.0000,1.0000] | 0.89 |
| Intercept | 0.000 | [0.0002,0.0003] | <0.001 |
| *Number of observations* | *424,212* |  |  |

**5a.ii ICU mortality multivariable regression model: hypothermia vs. normothermia (SOFA2)**

|  | OR | 95% CI | P-value |
| --- | --- | --- | --- |
| Hypothermia | 1.513 | [1.4201,1.6125] | <0.001 |
| Age | 1.016 | [1.0133,1.0178] | <0.001 |
| Gender |  |  |  |
| Male | 0.900 | [0.8445,0.9596] | 0.001 |
| SOFA2 | 1.428 | [1.4134,1.4427] | <0.001 |
| Ventilation on ICU day 1 | 5.448 | [5.0112,5.9236] | <0.001 |
| Frailty Score |  |  |  |
| 2 | 0.957 | [0.7681,1.1933] | 0.70 |
| 3 | 0.954 | [0.7684,1.1838] | 0.67 |
| 4 | 1.073 | [0.8596,1.3395] | 0.53 |
| 5 | 1.075 | [0.8493,1.3602] | 0.55 |
| 6 | 1.393 | [1.0998,1.7640] | 0.006 |
| 7 | 1.391 | [1.0675,1.8113] | 0.02 |
| 8 | 2.348 | [1.5822,3.4853] | <0.001 |
| Missing | 1.188 | [0.9612,1.4672] | 0.11 |
| Treatment limitation | 3.375 | [3.0878,3.6881] | <0.001 |
| Elective Surgery |  |  |  |
| Yes | 0.316 | [0.2901,0.3434] | <0.001 |
| Unknown | 0.671 | [0.3133,1.4359] | 0.30 |
| Operative group |  |  |  |
| Gastrointestinal | (ref) |  |  |
| Neurological | 3.473 | [3.1791,3.7945] | <0.001 |
| Plastics & Ortho. | 0.799 | [0.6996,0.9132] | 0.001 |
| Thoracic | 0.872 | [0.7345,1.0356] | 0.19 |
| Vascular | 1.415 | [1.2768,1.5674] | <0.001 |
| Genitourinary | 0.458 | [0.3740,0.5612] | <0.001 |
| Trauma | 1.856 | [1.6738,2.0574] | <0.001 |
| Obst/Gyn | 0.239 | [0.1435,0.3993] | <0.001 |
| Endocrine | 0.676 | [0.3524,1.2967] | 0.24 |
| Other | 2.506 | [1.7182,3.6537] | <0.001 |
| Pre-ICU hours | 1.000 | [1.0000,1.0000] | 0.75 |
| Chronic Respiratory | 1.402 | [1.2433,1.5820] | 0.001 |
| Chronic Cardiovsacular | 0.909 | [0.8057,1.0253] | 0.12 |
| Chronic Liver | 0.613 | [0.5058,0.6889] | <0.001 |
| Chronic Renal | 0.590 | [0.5058,0.6889] | <0.001 |
| Malignancy | 1.303 | [1.1430,1.4843] | <0.001 |
| Immunosuppressed | 1.170 | [1.0325,1.3265] | 0.01 |
| Diabetes | 1.091 | [1.0092,1.1785] | 0.28 |
| Intercept | 0.000 | [0.0003,0.0005] | <0.001 |
| *Number of observations* | *334,562* |  |  |

**5b.i ICU mortality multivariable regression model: 1-degree increments (APACHE III-j)**

|  | OR | 95% CI | P-value |
| --- | --- | --- | --- |
| Lowest temperature (°C) |  |  |  |
| 36-36.9 | 0.708 | [0.6250,0.8030] | <0.001 |
| 35-35.9 | 0.910 | [0.8029,1.0311] | 0.14 |
| 34-34.9 | 1.434 | [1.2476,1.6473] | <0.001 |
| 33-33.9 | 2.030 | [1.6986,2.4264] | <0.001 |
| 32-32.9 | 2.992 | [2.2112,4.0473] | <0.001 |
| <32 | 1.648 | [1.1444,2.3718] | 0.007 |
| Gender |  |  |  |
| Male | 1.025 | [0.9669,1.0871] | 0.40 |
| Other | 0.463 | [0.0523,4.0949] | 0.49 |
| APACHE III-J (no temperature) | 1.053 | [1.0514,1.0541] | <0.001 |
| Ventilation on ICU day 1 | 5.393 | [5.0040,5.8123] | <0.001 |
| Frailty Score |  |  |  |
| 2 | 1.001 | [0.8159,1.2290] | 0.99 |
| 3 | 1.035 | [0.8490,1.2621] | 0.73 |
| 4 | 1.145 | [0.9357,1.4020] | 0.19 |
| 5 | 1.135 | [0.9173,1.4041] | 0.24 |
| 6 | 1.392 | [1.1254,1.7220] | 0.002 |
| 7 | 1.469 | [1.1597,1.8598] | 0.001 |
| 8 | 2.861 | [2.0470,3.9992] | <0.001 |
| Missing | 1.131 | [0.9308,1.3740] | 0.22 |
| Treatment limitation | 2.856 | [2.6400,3.0899] | <0.001 |
| Elective Surgery |  |  |  |
| Yes | 0.377 | [0.3486,0.4067] | <0.001 |
| Unknown | 0.842 | [0.4674,1.5187] | 0.57 |
| Operative group |  |  |  |
| Gastrointestinal | (ref) |  |  |
| Neurological | 2.879 | [2.6553,3.1224] | <0.001 |
| Plastics & Ortho. | 0.743 | [0.6601,0.8372] | <0.001 |
| Thoracic | 0.864 | [0.7387,1.0103] | 0.07 |
| Vascular | 1.940 | [1.7693,2.1268] | <0.001 |
| Genitourinary | 0.596 | [0.5009,0.7081] | <0.001 |
| Trauma | 1.938 | [1.7621,2.1308] | <0.001 |
| Obst/Gyn | 0.303 | [0.1924,0.4768] | <0.001 |
| Endocrine | 0.673 | [0.3803,1.1918] | 0.17 |
| Other | 2.243 | [1.6253,3.0955] | 0.03 |
| Pre ICU hours | 1.000 | [1.0000,1.0000] | 0.97 |
| Intercept | 0.000 | [0.0003,0.0004] | <0.001 |
| *Number of observations* | *424,212* |  |  |

**5b.ii ICU mortality multivariable regression model: 1-degree increments (SOFA2)**

|  | OR | 95% CI | P-value |
| --- | --- | --- | --- |
| Lowest temperature (°C) |  |  |  |
| 36-36.9 | 0.802 | [0.6990,0.9192] | 0.002 |
| 35-35.9 | 1.061 | [0.9258,1.2169] | 0.39 |
| 34-34.9 | 1.688 | [1.4510,1.9637] | <0.001 |
| 33-33.9 | 2.249 | [1.8529,2.7300] | <0.001 |
| 32-32.9 | 2.764 | [1.9982,3.8231] | <0.001 |
| <32 | 1.449 | [0.9569,2.1936] | 0.08 |
| Age | 1.015 | [1.0132,1.0177] | <0.001 |
| Gender |  |  |  |
| Male | 0.913 | [0.8559,0.9731] | 0.005 |
| SOFA2 | 1.419 | [1.4040,1.4333] | <0.001 |
| Ventilation on ICU day 1 | 5.182 | [4.7632,5.6378] | <0.001 |
| Frailty Score |  |  |  |
| 2 | 0.955 | [0.7653,1.1908] | 0.68 |
| 3 | 0.954 | [0.7680,1.1851] | 0.67 |
| 4 | 1.078 | [0.8632,1.3472] | 0.51 |
| 5 | 1.088 | [0.8593,1.3785] | 0.48 |
| 6 | 1.414 | [1.1154,1.7914] | 0.004 |
| 7 | 1.414 | [1.0849,1.8426] | 0.01 |
| 8 | 2.342 | [1.5772,3.4763] | <0.001 |
| Missing | 1.199 | [0.9694,1.4820] | 0.09 |
| Treatment limitation | 3.364 | [3.0772,3.6785] | <0.001 |
| Elective Surgery |  |  |  |
| Yes | 0.318 | [0.2920,0.3459] | <0.001 |
| Unknown | 0.655 | [0.3029,1.4172] | 0.28 |
| Operative group |  |  |  |
| Gastrointestinal |  |  |  |
| Neurological | 3.378 | [3.0909,3.6918] | <0.001 |
| Plastics & Ortho. | 0.797 | [0.6972,0.9103] | 0.001 |
| Thoracic | 0.864 | [0.7273,1.0263] | 0.09 |
| Vascular | 1.319 | [1.1890,1.4638] | <0.001 |
| Genitourinary | 0.457 | [0.3730,0.5596] | <0.001 |
| Trauma | 1.799 | [1.6214,1.9956] | <0.001 |
| Obst/Gyn | 0.243 | [0.1462,0.4052] | <0.001 |
| Endocrine | 0.674 | [0.3502,1.2972] | 0.124 |
| Other | 2.483 | [1.7023,3.6219] | <0.001 |
| Pre-ICU hours | 1.000 | [1.0000,1.0000] | 0.70 |
| Chronic Respiratory | 1.418 | [1.2573,1.6003] | <0.001 |
| Chronic Cardiovascular | 0.922 | [0.8169,1.0399] | 0.19 |
| Chronic Liver | 0.623 | [0.5343,0.7270] | <0.001 |
| Chronic Renal | 0.606 | [0.5188,0.7070] | <0.001 |
| Malignancy | 1.302 | [1.1420,1.4834] | <0.001 |
| Immunosuppressed | 1.177 | [1.0379,1.3337] | 0.01 |
| Diabetes | 1.104 | [1.0216,1.1936] | 0.12 |
| Intercept | 0.001 | [0.0004,0.0007] | <0.001 |
| *Number of observations* | *334,562* |  |  |

**6. Figure S1: 90 day hospital mortality, by 1-degree increment**

**7. Figure S2: Hospital mortality vs temperature (unadjusted spline)**

**8. Figure S3: Hospital mortality vs temperature (adjusted SOFA2 based model)**

**9. Participating Hospital Sites**

**Australian Capital Territory**

Calvary Hospital (Canberra) ICU

Calvary John James Hospital ICU

Canberra Hospital ICU

National Capital Private Hospital ICU

North Canberra Hospital ICU

**New South Wales**

Bankstown-Lidcombe Hospital ICU

Bathurst Base Hospital ICU

Blacktown Hospital ICU

Bowral Hospital HDU

Broken Hill Base Hospital & Health Services ICU

Calvary Mater Newcastle ICU

Campbelltown Hospital ICU

Coffs Harbour Health Campus ICU

Concord Hospital (Sydney) ICU

Dubbo Base Hospital ICU

Fairfield Hospital ICU

Gosford Hospital ICU

Gosford Private Hospital ICU

Goulburn Base Hospital ICU

Grafton Base Hospital ICU

Griffith Base Hospital ICU

Hornsby Ku-ring-gai Hospital ICU

Hurstville Private Hospital ICU

John Hunter Hospital ICU

Kareena Private Hospital ICU

Lingard Private Hospital ICU

Lismore Base Hospital ICU

Liverpool Hospital ICU

Macquarie University Private Hospital ICU

Maitland Hospital ICU

Maitland Private Hospital ICU

Manning Rural Referral Hospital ICU

Mater Private Hospital (Sydney) ICU

Nepean Hospital ICU

Nepean Private Hospital ICU

Newcastle Private Hospital ICU

North Shore Private Hospital ICU

Northern Beaches Hospital ICU

Norwest Private Hospital ICU

Orange Base Hospital ICU

Port Macquarie Base Hospital ICU

Prince of Wales Hospital ICU

Prince of Wales Private Hospital ICU

Royal North Shore Hospital ICU

Royal Prince Alfred Hospital ICU

Ryde Hospital and Community Health Services ICU

Shoalhaven Hospital ICU

South East Regional Hospital ICU

St George Hospital (Sydney) ICU

St George Private Hospital (Sydney) ICU

St Vincent's Hospital (Sydney) ICU

St Vincent's Private Hospital (Sydney) ICU

Sutherland Hospital & Community Health Services ICU

Sydney Adventist Hospital ICU

Sydney Southwest Private Hospital ICU

Tamworth Base Hospital ICU

The Chris O’Brien Lifehouse ICU

Tweed Heads District Hospital ICU

Wagga Wagga Base Hospital & District Health ICU

Westmead Hospital ICU

Westmead Private Hospital ICU

Wollongong Hospital ICU

Wollongong Private Hospital ICU

Wyong Hospital ICU

**Northern Territory**

Alice Springs Hospital ICU

Royal Darwin Hospital ICU

**Queensland**

Brisbane Private Hospital ICU

Buderim Private Hospital ICU

Bundaberg Base Hospital ICU

Caboolture Hospital HDU

Cairns Hospital ICU

Gold Coast Private Hospital ICU

Gold Coast University Hospital ICU

Greenslopes Private Hospital ICU

Hervey Bay Hospital ICU

Ipswich Hospital ICU

John Flynn Private Hospital ICU

Logan Hospital ICU

Mackay Base Hospital ICU

Mater Adults Hospital (Brisbane) ICU

Mater Health Services North Queensland ICU

Mater Private Hospital (Brisbane) ICU

Mount Isa Hospital ICU

Noosa Hospital ICU

North West Private Hospital ICU

Pindara Private Hospital ICU

Princess Alexandra Hospital ICU

Queen Elizabeth II Jubilee Hospital ICU

Redcliffe Hospital ICU

Robina Hospital ICU

Rockhampton Hospital ICU

Royal Brisbane and Women's Hospital ICU

St Andrew's Hospital Toowoomba ICU

St Andrew's Private Hospital (Ipswich) ICU

St Andrew's War Memorial Hospital ICU

St Vincent’s Private Hospital Northside ICU

St Vincent's Hospital (Toowoomba) ICU

Sunnybank Hospital ICU

Sunshine Coast University Hospital ICU

Sunshine Coast University Hospital ICU - Paeds

Sunshine Coast University Private Hospital ICU

The Prince Charles Hospital ICU

The Wesley Hospital ICU

Toowoomba Hospital ICU

The Townsville University Hospital ICU

**South Australia**

Ashford Community Hospital ICU

Calvary Adelaide Hospital ICU

Calvary North Adelaide Hospital ICU

Flinders Medical Centre ICU

Flinders Private Hospital ICU

Lyell McEwin Hospital ICU

Royal Adelaide Hospital ICU

St Andrew's Hospital (Adelaide) ICU

The Memorial Hospital (Adelaide) ICU

The Queen Elizabeth (Adelaide) ICU

Western Hospital (SA)

Women's and Children's Hospital PICU

**Tasmania**

Calvary Hospital (Lenah Valley) ICU

Launceston General Hospital ICU

North West Regional Hospital (Burnie) ICU

Royal Hobart Hospital ICU

**Victoria**

Albury Wodonga Health ICU

Alfred Hospital ICU

Angliss Hospital ICU

Austin Hospital ICU

Ballarat Health Services ICU

Bendigo Health Care Group ICU

Box Hill Hospital ICU

Cabrini Hospital ICU

Casey Hospital ICU

Central Gippsland Health Service ICU

Dandenong Hospital ICU

Echuca Regional Hospital HDU

Epworth Eastern Private Hospital ICU

Epworth Freemasons Hospital ICU

Epworth Geelong ICU

Epworth Hospital (Richmond) ICU

Footscray Hospital ICU

Frankston Hospital ICU

Goulburn Valley Health ICU

Grampians Health Horsham ICU

Holmesglen Private Hospital ICU

John Fawkner Hospital ICU

Knox Private Hospital ICU

Latrobe Regional Hospital ICU

Maroondah Hospital ICU

Melbourne Private Hospital ICU

Mildura Base Hospital ICU

Monash Medical Centre-Clayton Campus ICU

Mulgrave Private Hospital ICU

Northeast Health Wangaratta ICU

Peninsula Private Hospital ICU

Royal Melbourne Hospital ICU

South West Healthcare (Warrnambool) ICU

St John Of God Hospital (Berwick) ICU

St John Of God Hospital (Ballarat) ICU

St John of God Hospital (Bendigo) ICU

St John Of God Hospital (Geelong) ICU

St Vincent's Hospital (Melbourne) ICU

St Vincent's Private Hospital Fitzroy ICU

Sunshine Hospital ICU

The Bays Hospital ICU

The Northern Hospital ICU

University Hospital Geelong ICU

Warringal Private Hospital ICU

Werribee Mercy Hospital ICU

Western District Health Service (Hamilton) ICU

**Western Australia**

Armadale Health Service ICU

Bunbury Regional Hospital ICU

Fiona Stanley Hospital ICU

Hollywood Private Hospital ICU

Joondalup Health Campus ICU

Mount Hospital ICU

Rockingham General Hospital ICU

Royal Perth Hospital ICU

Sir Charles Gairdner Hospital ICU

St John Of God Health Care (Subiaco) ICU

St John Of God Hospital (Murdoch) ICU

St John of God Midland Public & Private ICU

**New Zealand**

Auckland City Hospital CV ICU

Auckland City Hospital DCCM

Braemar Hospital SCU

Christchurch Hospital ICU

Dunedin Hospital ICU

Hawkes Bay Hospital ICU

Hutt Hospital ICU

Middlemore Hospital ICU

Nelson Hospital ICU

North Shore Hospital ICU

Rotorua Hospital ICU

Taranaki Health ICU

Tauranga Hospital ICU

Timaru Hospital ICU

Waikato Hospital ICU

Wairau Hospital ICU

Wellington Hospital ICU

Whakatane Hospital ICU

Whangarei Area Hospital, Northland Health
